# Synthesis and active manipulation of magnetic liquid beads

**DOI:** 10.1007/s10544-024-00708-z

**Published:** 2024-05-06

**Authors:** Ajeet Singh Yadav, Fariba Malekpour Galogahi, Aditya Vashi, Du Tuan Tran, Gregor S. Kijanka, Haotian Cha, Kamalalayam Rajan Sreejith, Nam-Trung Nguyen

**Affiliations:** Queensland Micro- and Nanotechnology Centre, Griffith University, 170 Kessels Road, Nathan, QLD 4111 Australia

**Keywords:** Magnetic liquid beads, Active sorting, Microfluidic device, Drug delivery, Self-assembly

## Abstract

We report the fabrication and characterisation of magnetic liquid beads with a solid magnetic shell and liquid core using microfluidic techniques. The liquid beads consist of a fluorinated oil core and a polymer shell with magnetite particles. The beads are generated in a flow-focusing polydimethylsiloxane (PDMS) device and cured by photo polymerisation. We investigated the response of the liquid beads to an external magnetic field by characterising their motion towards a permanent magnet. Magnetic sorting of liquid beads in a channel was achieved with 90% efficiency. The results show that the liquid beads can be controlled magnetically and have potential applications in digital microfluidics including nucleic acid amplification, drug delivery, cell culture, sensing, and tissue engineering. The present paper also discusses the magnetophoretic behaviour of the liquid bead by varying its mass and magnetite concentration in the shell. We also demonstrated the two-dimensional self-assembly of magnetic liquid beads for potential use in digital polymerase chain reaction and digital loop mediated isothermal amplification.

**Graphical abstract:**

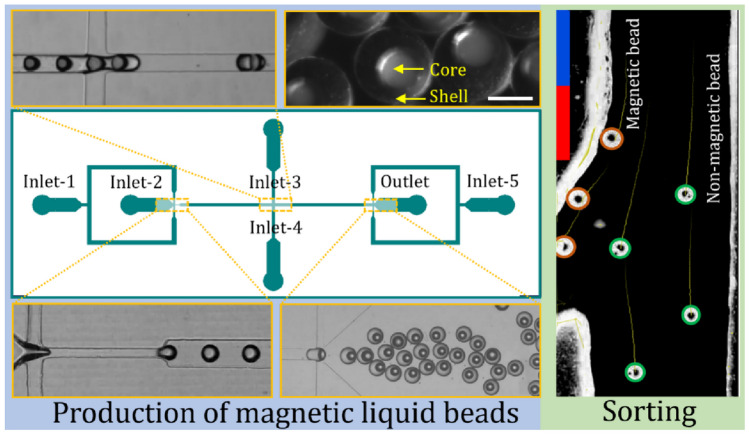

**Supplementary Information:**

The online version contains supplementary material available at 10.1007/s10544-024-00708-z.

## Introduction

Since its inception, digital microfluidics has gained wide popularity and acceptance. High precision, lower reagents requirement, ease of handling and low cost are some of the features leading to the wide acceptance of this technology. Liquid droplets, liquid marbles and liquid beads are the three major digital microfluidic platforms (Galogahi et al. 2021; Ooi et al. 2016). Liquid beads are core-shell particles with a solid shell and a liquid core. Liquid beads differ from liquid marbles (Aussillous and Quéré 2006) by their shell composition. Liquid beads have a solid shell, whereas liquid marbles have a soft powdered coating as shell. The shell material of liquid beads can be either organic polymers or organic-inorganic composites (Yadav et al. 2023; Yang et al. 2013). Liquid beads have gained increasing attention due to their distinctive core and shell structure enabling unique applications. With a liquid core encapsulated by a solid shell, liquid beads serve as reservoirs for controlled release of therapeutic agents in cell therapy, tissue regeneration, and provide three-dimensional (3D) cell culture environments for research and medical applications (Liu et al. 2021; Sun et al. 2018). In our previous works, we also demonstrated culturing microalgae (Tran et al. 2024) and amplification of deoxyribonucleic acid (DNA) (Yadav et al. 2023) in liquid beads. In addition, these beads are also useful in food processing (Wu et al. 2017), cosmetics (Foglio Bonda et al. 2020), and digital microfluidics (Kong et al. 2013; Wu et al. 2023). Specific examples are transportation and controlled release of substances such as pharmaceuticals (Phan et al. 2022), nutrients (Liu et al. 2024), and cells (Tan and Takeuchi 2007). These applications require a shell to protect the core from its surroundings, preventing contamination. The solid shell provides physical support to the core while ensuring there is no unintentional release to its surroundings. Considering these constraints, the shells can be designed to respond to a particular stimulus in one or several stages. The reported stimuli are electric field (Gao et al. 2021), a magnetic field (Spoială et al. 2021), light (Amoli-Diva et al. 2018), heat (Kurzhals et al. 2015), pH (Su et al. 2018), acoustic waves (Leibacher et al. 2014), and surface tension (Singha et al. 2019).

Microfluidic technique has been emerging as a flexible and effective method for generating emulsion and double emulsion or core–shell droplets, serving as a template to create liquid beads (Murshed et al. 2009; Tan et al. 2008). The compact and cost-effective nature of microfluidics offers substantial advantages over traditional methods (Galogahi et al. 2020). This technology allows for handling small fluid volumes in microchannels, facilitating accurate control over the core droplet size and shell thickness. Additionally, it yields droplets with a narrow size distribution at regular intervals (Yamagishi et al. 1996). To date, numerous studies have focused on encapsulating analytes within a single emulsion (Agresti et al. 2010; Wen et al. 2016). However, only a few works have been reported on double emulsion core-shell liquid beads (Malekpour Galogahi et al. 2023). The reported methods often include diverse undesirable components, necessitating subsequent purification (Huang et al. 2017). Active sorting allows for a more precise and specific selection of liquid beads based on their tailorable properties. Isolated core-shell structure enables enhanced sorting efficiency. Solid polymer shells are well suited as they can embed materials with specific stimuli responses. For instance, Magnetite and gold nano/microparticles can potentially be used for sorting, assembly, and triggered release of liquid beads. Furthermore, active sorting is easily automated and integrated into a larger system, suitable for high-throughput applications.

Separation of sensitive biological samples face many challenges. For instance, sorting cells based on their intrinsic properties may result in damage caused by shear stress during centrifugation, membrane pressure in filtration, or Joule heating in electrophoresis. Encapsulating biological sample with a magnetic shell offers advantages such as high specificity, low risk of cell damage and short sorting time. Magnetophoretic manipulation is independent of pH and ionic concentration of the solution (Chung et al. 2016; Fulcrand et al. 2011). Magnetic manipulation is relatively inexpensive compared to other active techniques, and suitable for industrial scale. Other advantages are their non-invasive nature and compatibility with microfluidics.

To our best knowledge, no work to date reports the synthesis and active manipulation of liquid beads with a solid magnetic shell and a liquid core. The magnetic solid shell is the key feature for applications requiring contamination-free active manipulation such as nucleic acid amplification. The present paper reports the synthesis, characterisation, and active manipulation of liquid beads with a magnetic solid shell and a liquid core. Subsequently we demonstrate a two-dimensional (2D) self-assembly of magnetic liquid beads with potential nucleic acid amplification techniques such as polymerase chain reaction (PCR) (Sreejith et al. 2020) and loop mediated isothermal amplification (LAMP) (Sreejith et al. 2021).

## Materials and methods

### Materials

Silicon wafers were acquired from IBD Technologies Ltd. (Wiltshire, UK). Photoresist SU-8 3050 was sourced from MicroChem Corp (Westborough, USA). Polydimethylsiloxane (PDMS) prepolymer and the associated curing agent (Sylgard 184) were procured from Dow Corning (Midland, MI, USA). Poly (vinyl alcohol) (PVA) with an 87–90% hydrolysis rate and an average molecular weight ranging from 30,000 to 70,000 was obtained from Sigma-Aldrich.

Spherical magnetite particles (Sigma-Aldrich, Iron II/III oxide) had a diameter of less than 5 μm and a density of approximately 5 × 10^3^ kg/m^3^. The shell liquid (polymer phase) was prepared by mixing 0.06 g of ethyl-4(dimethylamino)benzoate (Merck), 0.05 g of camphorquinone (Merck), 10 g of trimethylolpropane trimethacrylate (TMPTMA, Merck) and magnetite particles of concentrations of 1% and 2% by weight. The surface tension, viscosity, and density of this polymer solution were measured as σ_polymer_ = 0.032 N/m, µ_polymer_ = 0.042 Pa.s, and ρ_polymer_ = 1.07 × 10^3^ kg/m^3^ respectively (Takei et al. 2019).

Fluorinated oil (HFE Novec 7500 from 3 M) was selected as the core liquid. It has a surface tension of σ_HFE_ = 0.015 N/m, a viscosity of µ_HFE_ = 1.31 × 10^−3^ Pa.s, a density of ρ_HFE_ = 1.63 × 10^3^ kg/m^3^, and a boiling point of 128 °C (Rausch et al. 2015). For the outer continuous phase, an aqueous solution containing 50% glycerol (Chemsupply) in water (Milli-Q) was employed. This solution has a surface tension of σ_sol_ = 0.067 N/m, a viscosity of µ_sol_ = 6 × 10^−3^ Pa.s, and a density of ρ_sol_ = 1.12 × 10^3^ kg/m^3^ (Gregory 1991).

### Microfluidic synthesis of magnetic liquid beads

**Fig. 1:**
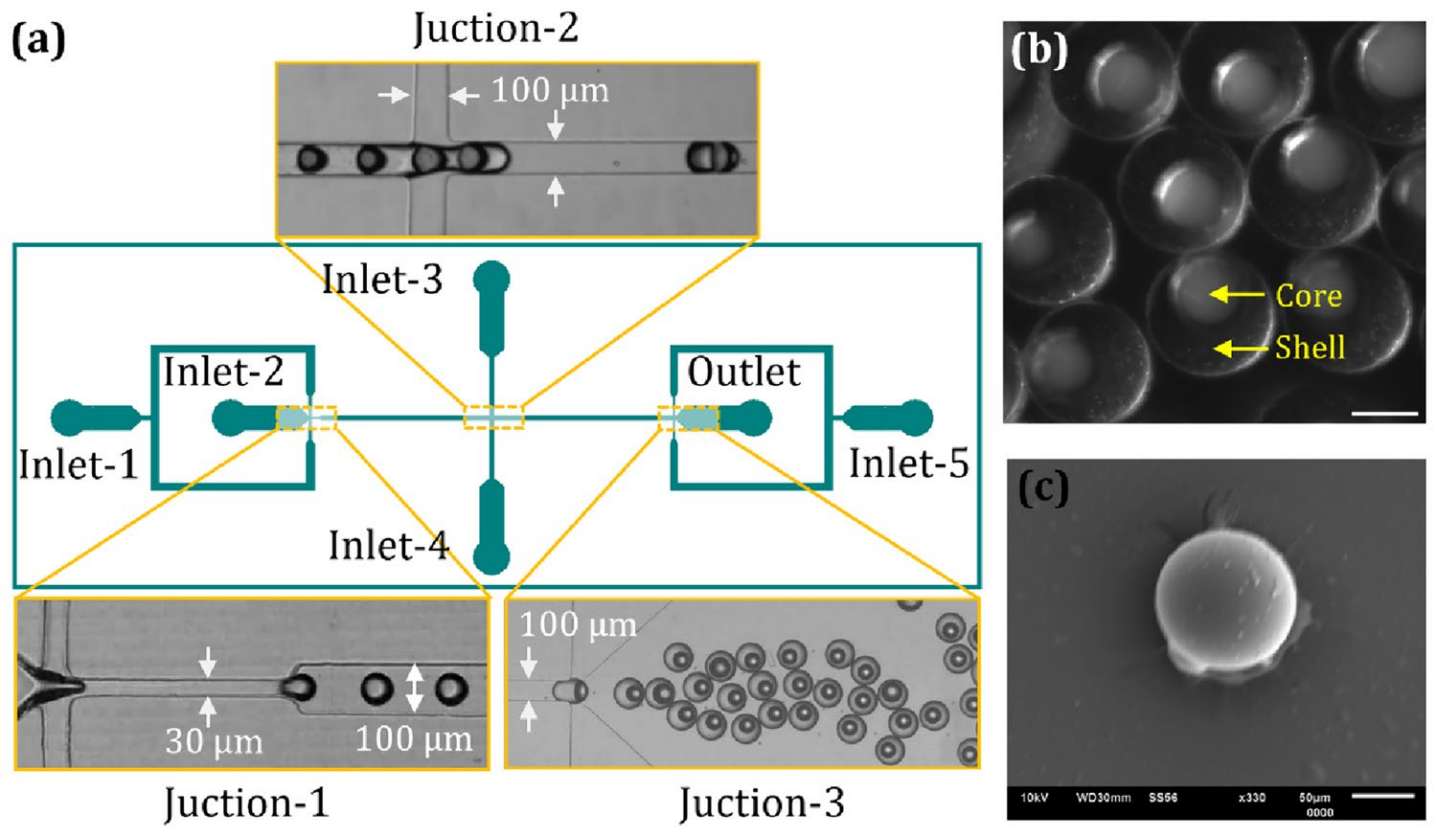
Fabrication of magnetic liquid beads. **a** The microfluidic device for generating core–shell droplets with an oil core. Inlet 1 introduces HFE 7500 oil, Inlet 2 introduces magnetite particles suspended in TMPTMA, and Inlets 3, 4, and 5 introduce an aqueous solution with 50% v/v glycerol and 0.06 mM Tween 20. The oil core droplets form at the first junction and are then directed to the second junction, where the shell envelops the core. A spacer fluid introduced at the third junction serves as a preventive measure against the accidental merging of core–shell droplets. Images of the resulting magnetic liquid beads captured through **b** optical microscope and **c** scanning electron microscope (SEM). Scale bar is 50 μm

A polydimethylsiloxane (PDMS) based microfluidic device was fabricated following our previous protocol (Galogahi et al. 2021, 2022). The process started with designing a mask with CleWin (WieWeb Software, The Netherlands). The design was then printed on a transparent film. A layer of SU-8 3050 (MicroChem) was spin-coated on a 4-inch silicon wafer. This layer was subsequently patterned with photolithography and annealing to create the mould for the microchannels. The microchannels were 100 μm wide and 120 μm deep with a constriction width of 30 μm, Fig. 1a.

The microfluidic device was fabricated using soft lithography. A degassed mixture of PDMS base and curing agent in a 10:1 ratio was poured onto the SU-8 mould and cured for 1 h at 75 °C. Once cured, the PDMS part was carefully peeled off. Inlets and outlets were created using a biopsy puncher. Finally, the PDMS device was bonded onto a glass substrate after treating both PDMS and glass substrate in an oxygen plasma cleaner (PDC-32G-2, Harrick Plasma).

The flow rates of the inlet fluids were regulated by a syringe pump (NEM-B101-03 A, CETONI GmbH, Germany). The core and shell fluids were injected through the first and second inlets, while the third and fourth inlets delivered the continuous phase, Fig. 1a. The core liquid flow rate was 30 µL/h, while the flow rates of the shell and the continuous phase was 100 and 400 µL/h, respectively. A spacer fluid was introduced through the last inlet at a flow rate of 500 µL/h to prevent unintended merging of core-shell droplets.

The core phase comprised HFE 7500 oil, while the shell phase consisted of TMPTMA mixed with ethyl 4-(dimethylamino) benzoate, camphorquinone, and magnetite particles (1 wt% and 2 wt%). Higher concentration of magnetite particles blocked the narrowest 30 μm-wide channel in the device. Prior to blending, particles were treated with sodium dodecyl sulphate (SDS) to minimise agglomeration. The excess surfactant was removed by rinsing with pH-11 NaOH solution. Subsequently, the washed particles were dispersed into Isopropyl alcohol and mixed to 2% by weight with the polymer (Choi et al. 2015; Tierno et al. 2005). The mixture was stirred mechanically for uniformity. The continuous phase and spacer fluid is an aqueous solution containing 50% v/v glycerol and 0.06 mM Tween 20.

To generate the core–shell droplets, the core phase flowed in the first inlet, where it comes in contact with the shell phase introduced through the second inlet at the first junction. Subsequently, the shell phase encapsulates the core droplets at the second junction to form the core-shell structure of the magnetic liquid bead. To disperse the shell phase, the continuous phase was introduced through the third and fourth inlets. The core–shell droplets then move to the third junction, where a spacer phase was added to prevent coalescence. Finally, the formed droplets exit the device, where they are collected in a Petri dish sitting on a gently oscillating platform. Following collection, the resulting core–shell droplets were exposed to a 24-W blue light (450–490 nm) source for a duration of 20 min to harden and form the liquid beads with oil core, Fig. 1b and c. Occasionally, during bead collection or the curing process, some beads may lose their core, resulting in solid beads. The bead size closely resembles that of core-shell beads. However, after analysing the density of HFE and TMPTMA and their volume within the bead, we discovered that solid beads were 5% lighter than their core-shell counterparts after the removal of the 50-µm HFE core, Supplementary Table S1.

### Microfluidic device for in-channel sorting of magnetic liquid beads

A separate PDMS sorting device was made utilising a 1-mm thick PMMA sheet as a mould with the design drawn using CoralDRAW. The channels were cut into the sheet using a laser cutter (Rayjet 300, Trotec). Subsequently, the cut pattern was glued to a glass slide and placed in a Petri dish. To make the device, a mixture of PDMS and the curing agent was poured onto the PMMA mould and cured for 1 h at 75 °C. Once cured, the PDMS replica was delicately peeled off. The inlets and outlets were punched. The PDMS part was then bonded to a glass slide, completing the device for in-channel sorting of magnetic liquid beads.

**Fig. 2:**
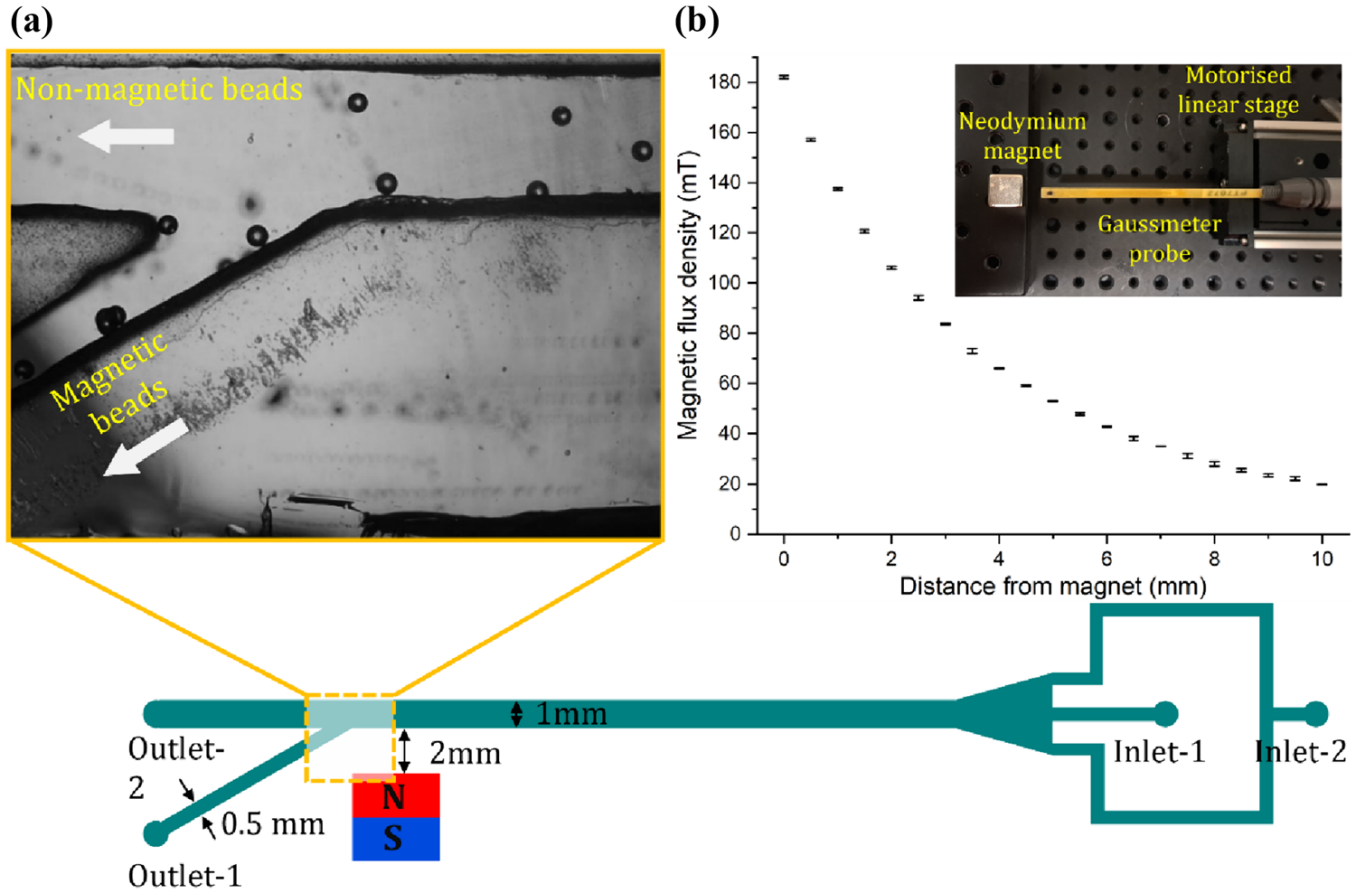
In-channel sorting of liquid beads: **a** Schematic of microfluidic channel demonstrating sorting of magnetic and non-magnetic liquid beads; **b** Magnetic flux density as function of distance from of the permanent magnet. The inset photograph shows the experimental setup

This microfluidic device consists of two straight channel sections with dimensions of 35 mm × 1 mm and 15 mm × 0.5 mm (length × width), respectively. Additionally, a flow-focusing configuration was introduced at the inlet to focus the beads to a single line for sorting. The height of the channel was determined by the 1-mm thick PMMA sheet. A neodymium permanent magnet (13.5 × 13.5 × 13.5 mm^3^) was placed on one side of the straight section at a lateral distance of 2 mm as depicted in Fig. 2a. The device was not fabricated by the standard photolithography and soft lithography techniques due to the relatively large channel height of 1 mm, which is not easily achievable with photo lithography of SU-8. This relatively large channel height of 1 mm was necessary to avoid channel blockage caused by liquid beads. The magnetic flux density (*B*) of the magnet (neodymium, 13.5 × 13.5 × 13.5 mm^3^) was measured using a handheld gaussmeter (GM07, measurement range: 0 to 3 T, Hirst Magnetic Instruments Ltd, UK). The magnet was vertically mounted to a PMMA holder, positioned on a linear stage, Fig. 2b (inset). Initially, the probe makes contact with the magnet to obtain the maximum reading. Subsequently, the linear stage was programmed to incrementally move 0.5 mm away from the probe for each data point. Figure 2b illustrates the measured magnetic flux densities as a function of the distance between the gaussmeter probe and the magnet.

## Results and discussions

### Manipulation of floating magnetic liquid beads

**Fig. 3:**
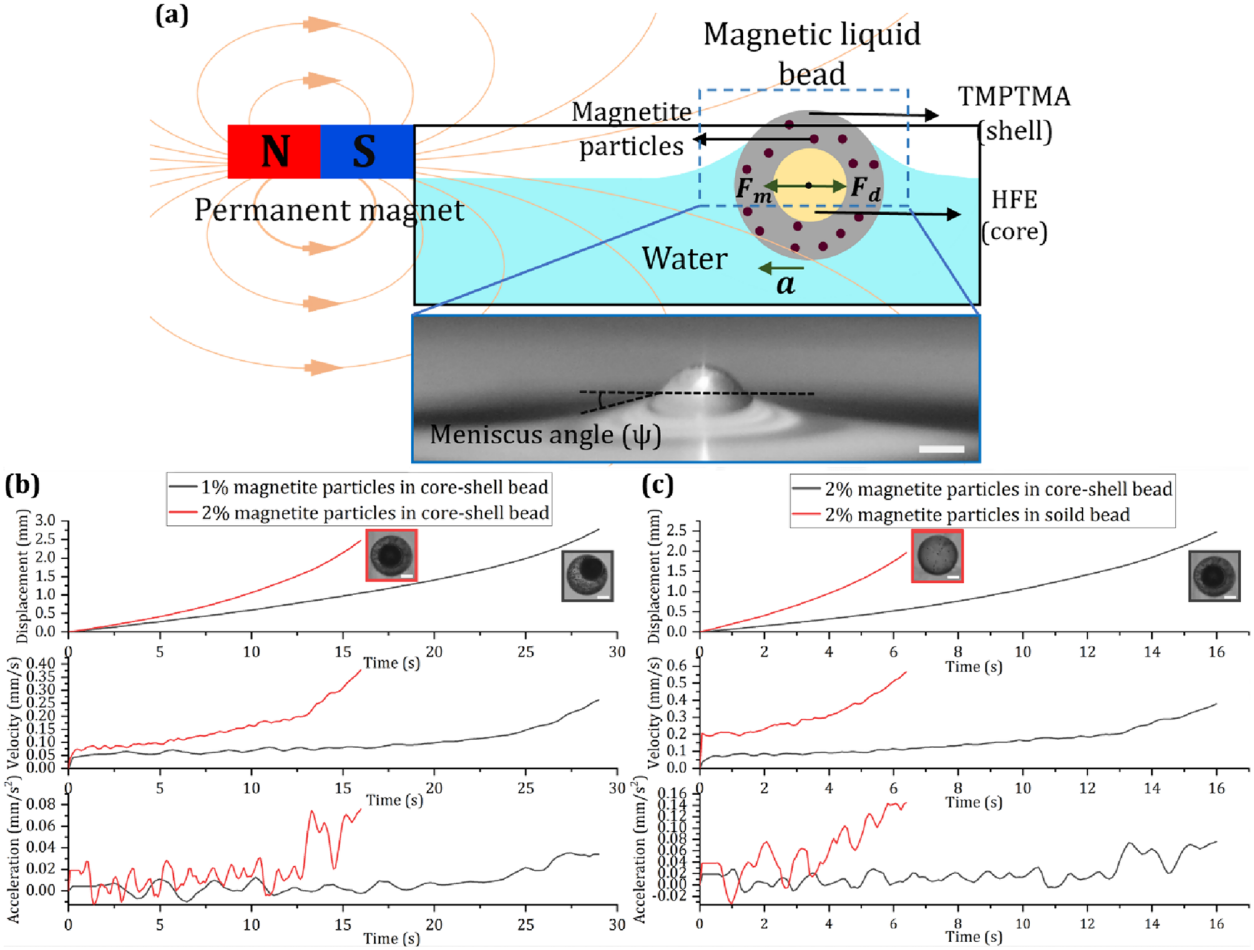
Manipulation of floating magnetic liquid beads: **a** Horizontal forces acting on the floating liquid bead (magnetic force $F_m$ and drag force $F_d$).  The separation between the floating bead and the magnet measures 3 mm. The magnetite particle concentration was varied at 1% and 2% (wt). Scale 50 μm; **b** Displacement, velocity, and acceleration of core-shell beads with 1% and 2% (wt) of magnetite particles in the shell; **c** Displacement, velocity, and acceleration of 2% (wt) magnetite particle containing core-shell and solid beads. Scale bar is 25 μm

Initial experiments were carried out to demonstrate the manipulation of floating magnetic liquid beads in a magnetic field. We investigated core-shell liquid beads and solid beads with different weight percentages of magnetite particles. The solid beads were 5% lighter than core-shell beads, the mass contribution of magnetite particles was neglected. Figure 3a shows the experimental setup that comprises of a permanent magnet and a container with water. The magnet’s height was set to match the water level in the container. The entire setup was mounted on a microscope (Nikon Eclipse Ti1) for real-time monitoring. A liquid bead was carefully positioned on the water surface, ensuring it remained sufficiently distant from the magnet (5 mm) to avoid interference. The magnet was then gradually moved closer to within 3 mm of the particle using a linear stage until the floating magnetic bead starts to accelerate towards the magnet. The motion of the bead was monitored and recorded using a DSLR camera (Nikon D7500) attached to the microscope. The images were extracted from the recorded videos, and further analysis of the images was conducted using the TrackMate plugin within the ImageJ software (Ershov et al. 2022). The formed beads were collected in a Petri dish and positioned over the magnet to demonstrate 2D self-assembly. Images and videos were recorded to document the process.

When a liquid bead is placed carefully on the surface of a water-filled container, it floats at the water-air interface, Fig. 3a. Floating condition of the denser-than-water bead arises from the equilibrium between downward gravitational force, upward forces of buoyancy acting on the bead and surface tension of the water. Khaw et al. (2016) reported the application of magnetically actuated floating liquid marbles as a platform for digital microfluidics, investigating the forces and conditions essential for controlled movement. Our current study focuses on floating magnetic liquid beads, examining factors influencing their motion, such as magnetic flux density and particle concentration. Under the field of a permanent magnet, the force balance acting on the floating bead can be described as:

1 $${m}_{p}{a}_{p}{=F}_{m}+{F}_{d}$$

where ${m}_{p}$ and ${a}_{p}$ are mass and acceleration of the bead.

Assuming the surrounding medium to be diamagnetic, the driving magnetic force acting on a floating liquid bead is (Nguyen et al. 2010; Shevkoplyas et al. 2007).

2 $${F}_{m}=\frac{V\chi }{{\mu }_{o}}B\frac{dB}{dx}$$

where ${F}_{m}$ represents the magnetic force, $V$ denotes the volume of the magnetite, $\chi$ is the magnetic susceptibility of the floating bead, ${\mu }_{o}$ is the permeability of vacuum, and $B$ represents the magnetic flux density.

Drag force occurs when an object moves at a relative velocity with respect to its surrounding fluid. This force originates from the necessity to displace the fluid elements in the path of the moving object. The drag force acting on a partially submerged spherical particle is described by Stokes’ law:

3 $${F}_{d}=6\pi \beta \mu {r}_{p}{\upsilon }_{p}$$

where $\beta$ is correction factor for coefficient of friction, $\mu$ is the dynamic viscosity of fluid, ${r}_{p}$ and ${\upsilon }_{p}$ represents the radius and horizontal velocity of the bead respectively. As reported in our previous work, $\beta$ is dependent on the degree of perturbation in the liquid interface (Ooi et al. 2016). This perturbation, in turn, depends on both the meniscus angle (ψ) and the submerged depth of the liquid bead, Fig. 3a. The bead was assumed to be a solid spherical body which maintains its shape during the motion.

According to Eq. 2, the magnetic force increases with increasing volume of the magnetic shell, increasing flux density and flux density gradient of the magnetic field and the concentration of the magnetite particles in the shell (Khaw et al. 2016). According to this understanding, we developed two hypotheses: (i) Liquid beads with higher concentration (2% wt) of magnetite particles experience a higher magnetic force and acceleration. (ii) Assuming a uniform distribution of magnetite particles throughout the solid shell, the overall volume remains constant for all core-shell beads. In the case of a solid bead without a core, the volume available to magnetite particles increases. Thus, the solid beads experience a higher magnetic force and acceleration compared to a liquid bead with as shell of the same magnetite concentration.

Figure 3b depicts the results of experiments carried out to characterise the magnetic response of beads. The floating magnetic bead starts experiencing magnetic force when the permanent magnet was placed at a distance of 3 mm. We observed that the liquid beads with 2% (wt) magnetite particles experience more force compared to 1% (wt) counterparts. Thus, the acceleration of 2% (wt) magnetic beads is higher compared to its counterpart. These results proved our first hypothesis.

Figure 3c shows the results of the experiments to test the second hypothesis. Assuming a uniform distribution of magnetite particles throughout the TMPTMA material, the overall volume remains constant for all core-shell beads. In the case of a solid bead without a core, the volume available to magnetite particles increases by 13%, Supplementary Table S2. Additionally, the solid bead is 5% lighter than the core-shell (values were calculated from the mass density and volume values of TMPTMA and HFE oil core liquid). Thus, the solid beads experience larger force compared to liquid beads of the same magnetite concentration. The results clearly shows that the 2% (wt) solid beads experienced higher force compared to 2% (wt) liquid beads and accelerated more than its counterpart. Supplementary Fig. S3, compares the displacement, velocity, and acceleration of various floating beads under the effect of external magnetic field.

### Sorting of in-channel magnetic liquid beads

Magnetic and non-magnetic liquid beads synthesised by the microfluidic device described in Section 2.2 were collected after curing and then redispersed in water for in-channel sorting experiments. The beads were transferred to a glass syringe through Polytetrafluoroethylene (PTFE) tubing. A 10% Poly (vinyl alcohol) (PVA) solution was flushed into the syringe and tubing for 10 min, followed by a 15-minute baking process at 100 °C. This process was repeated three times to achieve a desired level of hydrophilicity and prevented the beads from adhering to the syringe and tubing walls.

We introduced two streams into a central channel: one containing a mixture of magnetic and non-magnetic beads dispersed into water, and the other containing water serving as a buffer. The flow in the main channel was separated into outlet 1 and outlet 2 downstream, Fig. 2a. The optimal flow rates for the buffer and the beads dispersed medium were 8 ml/hr and 4 ml/hr, respectively. The trajectories of 100-µm magnetic and non-magnetic beads were observed within the microfluidic device. The deflection of beads depends on the balance between the magnetic force and the hydrodynamic drag force. Outlet 1 (0.5 × 1 mm) branches out at a 30° angle from the straight outlet 2 (1 × 1 mm). This design ensures that magnetic beads, affected by the magnetic field deviate from the straight path and exit from outlet 1, while non-magnetic beads continue along the straight path and exit from outlet 2, Supplementary Video S4. To evaluate sorting efficiency, bead motion was monitored using the ImageJ plugin Track Mate. Sorting experiments were carried out with a permanent magnet to ensure that the beads collected from outlet 1 were magnetic and those from outlet 2 were non- magnetic. The collected beads from outlets 1 and 2 were dispersed in water in two different transparent plastic collection tubes. The tubes were shaken well to achieve a reasonably homogenous dispersion.

**Fig. 4:**
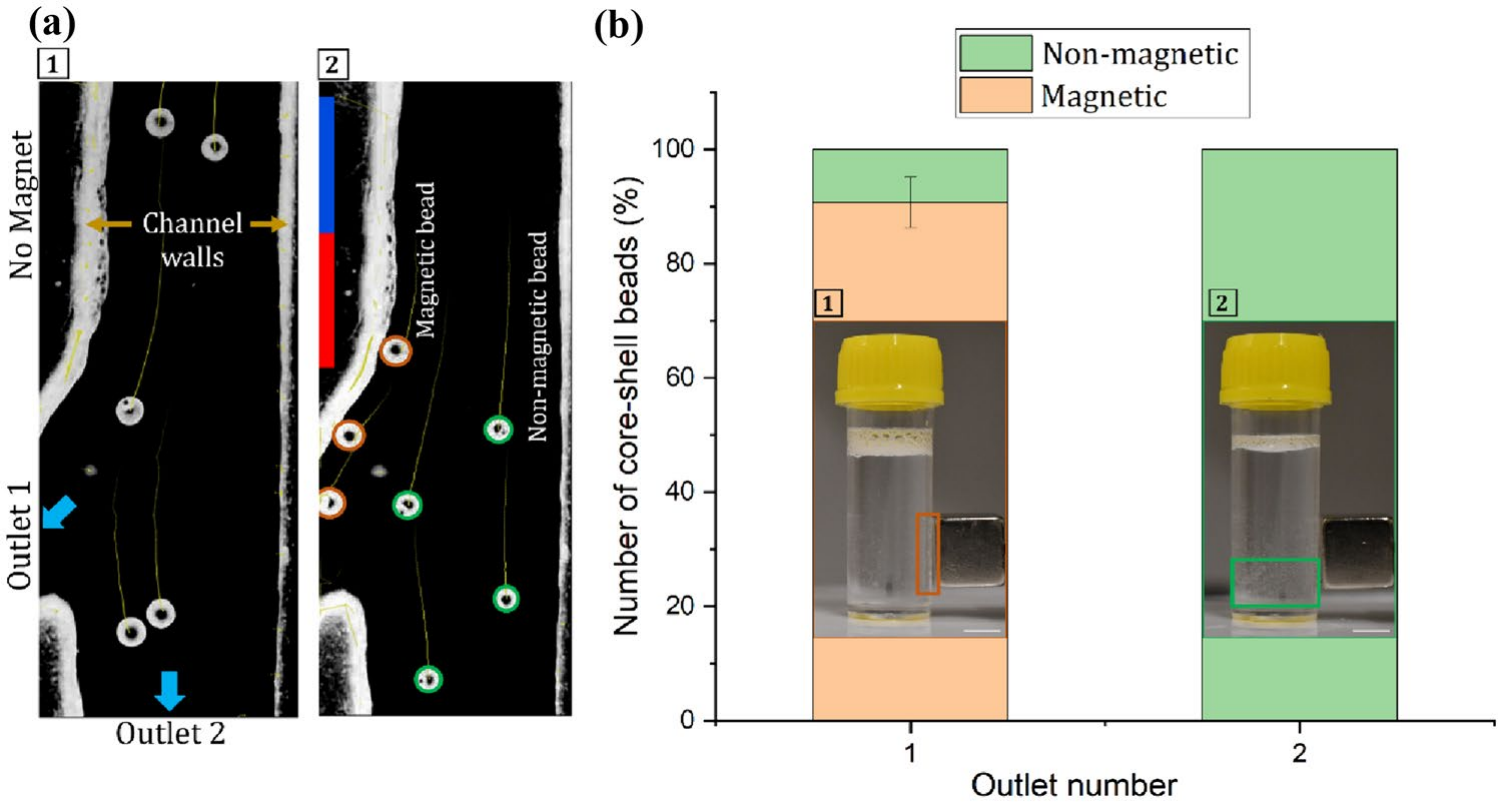
Sorting magnetic of non-magnetic liquid beads. **a** Trajectories of beads within a microfluidic channel with [1] absence and [2] presence of a magnet; **b** Bar graph representing sorting efficiency. [1] Magnetic beads liquid accumulating on the wall of the container near the external magnet. [2] Non-magnetic liquid beads from outlet 2 settle to the bottom of the container. Scale bar is 10 mm

Figure 4 depicts the results of the sorting experiment. The trajectories of beads over the last 10 frames are illustrated by the tails. All liquid beads depicted in Fig. 4a[1] maintain straight trajectories and exit solely through outlet 2 in the absence of the magnet. In Fig. 4a[2], magnetic liquid beads exhibit deviation from a straight path, move close to the channel wall next to the magnet, and travel along the wall under the magnetic field. The beads exiting from outlet 1 are therefore magnetic, while those exiting from outlet 2 are non-magnetic. About 10% beads exit from outlet 1 without showing lateral deflection, leading to sorting error. Conversely, none of the beads after deviating towards the channel wall exited from outlet 2. Figure 4b presents the outcomes after counting the magnetic and non-magnetic beads exiting from outlet 1 and 2. It shows that 90% of beads collected at outlet 1 were magnetic, while all beads collected at outlet 2 were non-magnetic. Figure 4b[1] and [2] show the beads collected from outlet 1 and 2, respectively. The beads were dispersed in water and stored in two separate collection tubes. A permanent magnet was placed on the container wall. We observed that the beads collected from outlet 1 was accumulated near the wall of the container where the magnet is placed. This proves that the beads collected from outlet 1 are magnetic. The nonmagnetic beads in the other container were not affected by external magnetic field and sedimented at the bottom of the container. Supplementary Videos S5 and S6 provides evidence of the response of beads collected in the tubes.

### Assembly of magnetic liquid beads

**Fig. 5:**
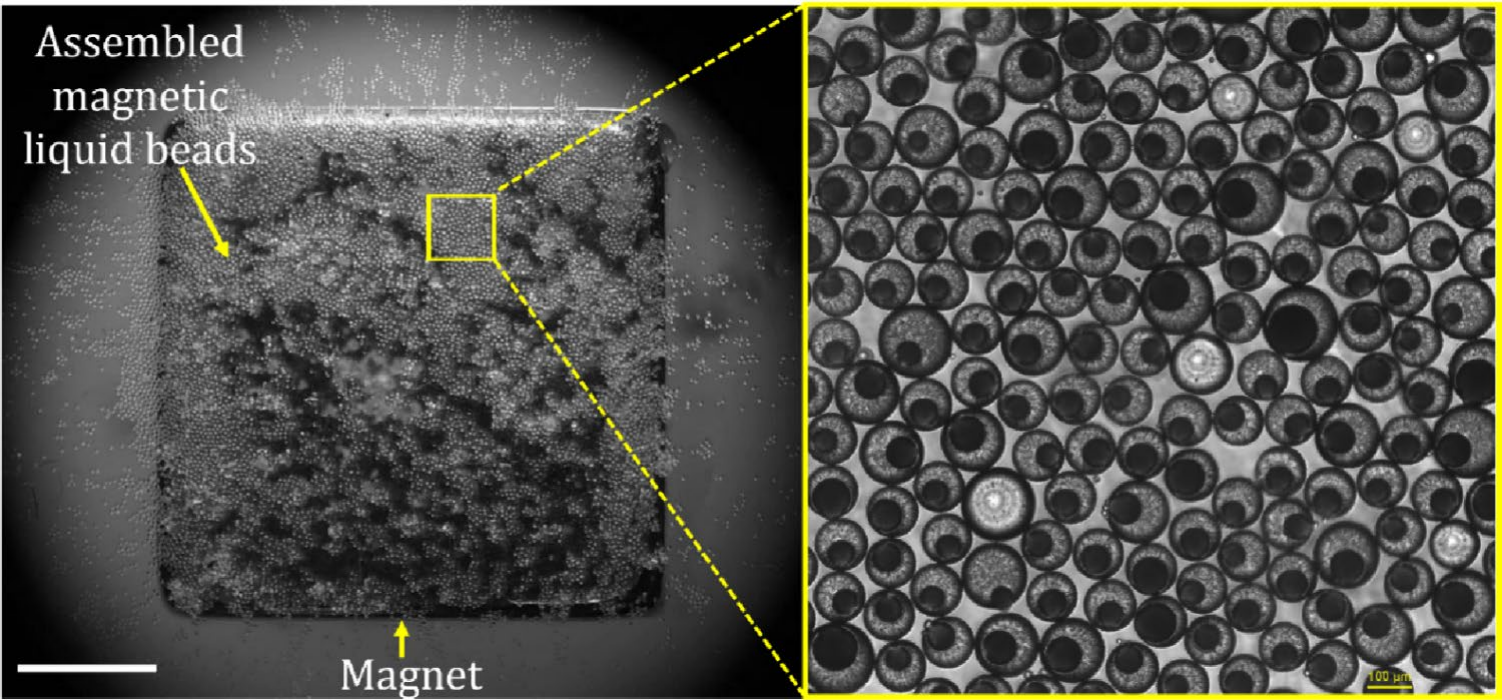
2D self-assembly of magnetic beads floating in water in a Petri dish with a permanent magnet placed under the dish. Scale is 5 mm

We further conducted an experiment to demonstrate the 2D self-assembly of the liquid beads which plays a significant role in nucleic acid amplification techniques such as digital polymerase chain reaction (dPCR) and digital loop mediated isothermal amplification (dLAMP). The magnetic liquid beads collected from outlet 1 were dispersed in water in a Petri dish. A neodymium magnet (13.5 × 13.5 × 13.5 mm^3^) was placed under the Petri dish. The whole assembly was kept under the microscope. The 2D self-assembly of the magnetic liquid beads was observed. Figure 5 shows the 2D self-assembled magnetic liquid beads. Supplementary Video S7 shows the self-assembly process. Being a highly efficient bioreactor for nucleic acid amplification, liquid beads have been attracting attention from the research community. The liquid beads discussed in this paper function as micro-reactors capable of encapsulating samples without contamination, while also being magnetically manipulable. It is highly desirable for the analytes present in the core not to be contaminated with magnetic particles, enhancing fluorescence signals following the successful amplification.

The two-dimensional (2D) self-assembly demonstrated here is also a highly desirable feature for dPCR and dLAMP as it enhances the accuracy by the reducing the scanning area. Magnetic liquid beads have the potential to be assembled into a grid-like configuration, mimicking commercially available PCR wells, all without the need for well plates. This will significantly contribute towards reducing the plastic wastage. Hence, we believe that the innovative method of synthesis, sorting, and 2D self-assembly of our magnetic liquid beads will notably impact the field of digital microfluidics.

## Conclusion

This paper reports the synthesis of magnetic liquid beads within a flow-focusing PDMS microfluidic device. Through the surface treatment of magnetite particles, we successfully achieved a stable and uniform blending with TMPTMA, resulting in homogenous beads with smooth surfaces and spherical shape. However, some of the beads lack cores and we termed them as solid beads. Subsequently, we investigated the behaviour of floating magnetic liquid beads. In the presence of a permanent magnet, the motion of magnetic beads was investigated. While the permanent magnet was fixed, the concentration of magnetite particles in the shell, its volume distribution and mass of the beads were varied. Next, we demonstrated sorting of magnetic and non-magnetic using a permanent magnet. In addition, our experiment demonstrated potential sorting of magnetic liquid beads from magnetic solid beads. However, this would require fine tuning the magnetic forces by incorporating more sophisticated magnet designs into microfluidic devices (Pamme et al. 2006; Watarai and Namba 2002). We achieved sorting efficiency of 90% that can also be improved by incorporating better flow focusing channel designs. The reduction in efficiency is attributed to the inhomogeneous distribution of magnetite particles in the beads. We achieved a throughput of 150–200 beads/min. Finally, we demonstrated the 2D self-assembly of magnetic liquid beads. We believe that the synthesis, active manipulation and self-assembly of liquid beads with solid magnetic shell will provide a contamination-free bioreactor for applications such as dPCR and dLAMP with higher accuracy in terms of fluorescence and related statistical analysis. Further research in this direction is currently underway.

### Supplementary Information

Below is the link to the electronic supplementary material.

Supplementary file1 (DOCX 241 kb)

Supplementary file2 (AVI 128000 kb)

Supplementary file3 (AVI 11300 kb)

Supplementary file4 (AVI 11600 kb)

Supplementary file5 (AVI 105000 kb)

## Data Availability

No datasets were generated or analysed during the current study.
