# Supplementary material for: Synthesis and active manipulation of magnetic liquid beads
Source: Biomed Microdevices. 2024 May 6;26(2):24. doi: 10.1007/s10544-024-00708-z (PMC11074228; doi:10.1007/s10544-024-00708-z)
Supplement: Supplementary file 1 — Supplementary file1 (DOCX 241 kb) [file 10544_2024_708_MOESM1_ESM.docx]

Synthesis and active manipulation of magnetic liquid beads

Ajeet Singh Yadav^1^, Fariba Malekpour Galogahi^1^, Aditya Vashi^1^, Du Tuan Tran^1^, Gregor S Kijanka^1^, Haotian Cha^1^, Kamalalayam Rajan Sreejith^1^ and Nam-Trung Nguyen^1, *^

^1^Queensland Micro- and Nanotechnology Centre, Griffith University, 170 Kessels Road, Nathan, QLD 4111, Australia.

^*^Corresponding author

**Table S1:** Mass ratio of liquid bead and solid bead

| Mass ratio of liquid bead and solid bead | |
| --- | --- |
| Density of TMPTMA (g/ cm^3^) | = 1.1 |
| Density of HFE 7500 oil(g/ cm^3^) | = 1.6 |
| Mass of solid bead(density$\boldsymbol{\times}$volume) (g) | = $\boldsymbol{5.75959\times}\boldsymbol{10}^{\boldsymbol{-7}}$ |
| Volume of solid bead (cm^3^) | = $\boldsymbol{5.23599\times}\boldsymbol{10}^{\boldsymbol{-7}}$ |
| Volume of shell (cm^3^) | = $\boldsymbol{4.58149\times}\boldsymbol{10}^{\boldsymbol{-7}}$ |
| Volume of core (cm^3^) | = $\boldsymbol{6.54498\times}\boldsymbol{10}^{\boldsymbol{-8}}$ |
| Mass of core-shell bead(density$\boldsymbol{\times}$volume) (g) | = $\boldsymbol{6.08684\times}\boldsymbol{10}^{\boldsymbol{-7}}$ |
| Mass Ratio(solid bead/liquid bead) | = 0.946236559 |

**Table S2:** Ratio of volumes available in solid and core-shell beads for magnetite particles

| Volume ratio of a solid and liquid beads | | |
| --- | --- | --- |
| Solid bead | Liquid bead | Volume Ratio |
| Radius outer (mm)= 0.05 | Radius outer (mm)= 0.05 | **Volume (solid): Volume (shell of the core-shell bead) = 0.875** |
| Radius inner (mm) = 0 | Radius inner (mm)= 0.025 |  |
| Volume(particle)(mm^3^) = 0.000523599 | Volume(shell)(mm^3^) =0.000458 |  |

**Figure S3:** Motion of magnetic liquid beds represented by (a) displacement versus distance from magnet, (b) velocity versus distance from magnet and (c) acceleration versus distance from magnet graphs.


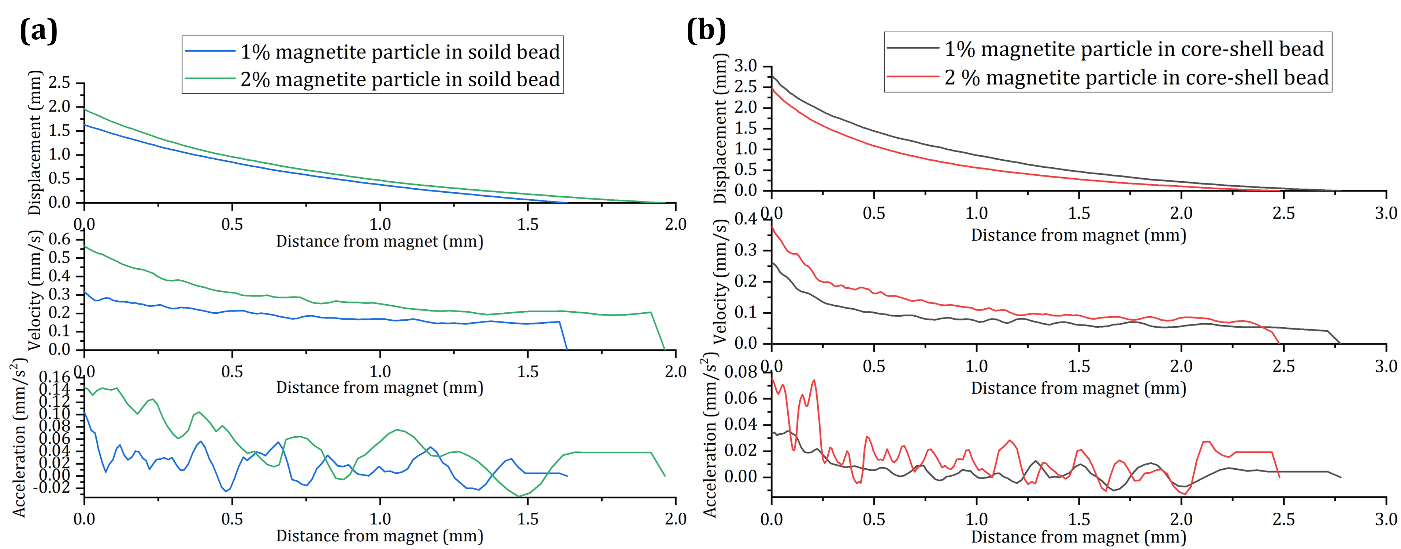


**Figure S3:** Motion of magnetic liquid beads: (a) Displacement, velocity, and acceleration versus distance from magnet for 1 and 2%(wt) magnetite particle containing solid beads [2]. (b) Displacement, velocity, and acceleration versus distance from magnet for 1 and 2%(wt) magnetite particle containing core-shell beads

Video S4: Video demonstrating sorting of magnetic liquid beads from non-magnetic beads.

## Video S5: Video showing response of beads collected from outlet 1 in presence of the magnet.

## Video S6: Video showing response of beads collected from outlet 2 in presence of the magnet.

## Video S7: Video showing 2D self-assembly of magnetic liquid beads in presence of magnet.
